# Supplementary material for: Neoadjuvant Therapy Is Associated with Improved Chemotherapy Delivery and Overall Survival Compared to Upfront Resection in Pancreatic Cancer without Increasing Perioperative Complications
Source: Cancers (Basel). 2022 Jan 26;14(3):609. doi: 10.3390/cancers14030609 (PMC8833799; doi:10.3390/cancers14030609)
Supplement: Supplementary file 1 [file cancers-14-00609-s001.zip › cancers-1543144-SI.pdf]

Supplementary Table S1. Comparison of OS, hospital length of stay, early and late perioperative complications, and vascular rates of patients who underwent neo-chemoRT+/- neoCHT or neoCHT alone.

	Neo-chemoRT +/- neoCHT		NeoCHT only		P value
	n	(%)	n	(%)	
**Total**	33		48		
**Overall Survival**	32.4 mos		27.2 mos		0.60
**Hospital LOS**					
Mean (SD)	17.3		15.1		0.70
Range	2 - 138		6-104		
**C-D Grade 3+ Complications**					
Yes	9	(28.1)	10	(20.8)	0.45
No	23	(71.9)	38	(79.2)	
**30-day Bile Leaks**					
Yes	3	(9.4)	1	(2.1)	0.15
No	29	(90.6)	46	(97.9)	
**Non-pancreatic/chylous/organ space infection**					
Yes	4	(12.5)	1	(2.1)	0.077
No	28	(87.5)	47	(97.9)	
**Post-operative wound disruption**					
Yes	9	(27.3)	9	(19.2)	0.39
No	24	(72.7)	38	(80.9)	
**Post-operative Sepsis**					
Yes	1	(3)	7	(14.9)	0.08
No	32	(97)	40	(85.1)	

**Late (>30 day) leak**					
Yes	1	(3.2)	5	(10.6)	0.23
No	30	(96.8)	42	(89.4)	
**30 day mortality**					
Yes	1	(3)	1	(2.1)	0.80
No	32	(97)	46	(97.9)	
**30 day readmission rate**					
Yes	10	(32.3)	11	(23.4)	0.39
No	21	(67.7)	35	(76.6)	
**30-90 day readmission rate**					
Yes	8	(25.8)	8	(17.0)	0.35
No	23	(74.2)	39	(83)	
**Any readmission (0-90 days)**					
Yes	13	(39.4)	18	(37.5)	0.86
No	20	(60.6)	30	(62.5)	
**Vascular resection**					
Yes	17	(54.8)	20	(43.5)	0.33
No	14	(45.2)	26	(56.5)	
